# FALCON: a software package for analysis of nestedness in bipartite networks

**DOI:** 10.12688/f1000research.4831.1

**Published:** 2014-08-06

**Authors:** Stephen J. Beckett, Chris A. Boulton, Hywel T. P. Williams

**Affiliations:** 1 College of Life and Environmental Sciences, University of Exeter, Exeter, EX4 4QE,, UK

## Abstract

Nestedness is a statistical measure used to interpret bipartite interaction data in several ecological and evolutionary contexts, e.g. biogeography (species-site relationships) and species interactions (plant-pollinator and host-parasite networks). Multiple methods have been used to evaluate nestedness, which differ in how the metrics for nestedness are determined. Furthermore, several different null models have been used to calculate statistical significance of nestedness scores. The profusion of measures and null models, many of which give conflicting results, is problematic for comparison of nestedness across different studies.

We developed the FALCON software package to allow easy and efficient comparison of nestedness scores and statistical significances for a given input network, using a selection of the more popular measures and null models from the current literature. FALCON currently includes six measures and five null models for nestedness in binary networks, and two measures and four null models for nestedness in weighted networks. The FALCON software is designed to be efficient and easy to use. FALCON code is offered in three languages (R, MATLAB, Octave) and is designed to be modular and extensible, enabling users to easily expand its functionality by adding further measures and null models. FALCON provides a robust methodology for comparing the strength and significance of nestedness in a given bipartite network using multiple measures and null models. It includes an “adaptive ensemble” method to reduce undersampling of the null distribution when calculating statistical significance. It can work with binary or weighted input networks. FALCON is a response to the proliferation of different nestedness measures and associated null models in the literature. It allows easy and efficient calculation of nestedness scores and statistical significances using different methods, enabling comparison of results from different studies and thereby supporting theoretical study of the causes and implications of nestedness in different biological contexts.

## Introduction

Nestedness is a statistical property of systems where two kinds of entity interact, which can be represented as bipartite networks. Originally used as a metric for species-site distributions ^1, 2^, nestedness has recently gathered much attention as a metric for bipartite species interaction networks, e.g. plant-pollinator mutualisms ^3, 4^ and host-virus interactions ^5– 7^. Various discussions have considered the sources of nestedness in such systems and its potential implications for ecological dynamics ^4, 8– 13^. However, it is unclear how to systematically compare results for different ecological datasets. Furthermore, nestedness is not restricted to ecological datasets, but is a generic property of any bipartite network. Thus, there is a need for measures of nestedness that are context-independent and do not depend on any particular (ecological) interpretation. Multiple methods for measuring nestedness have been used in different studies, along with multiple approaches to calculating statistical significance of the measured values. This provides a large number of ways in which nestedness could be evaluated ^14– 16^. Before theoretical investigations of the mechanisms of nestedness can be properly undertaken, robust measures and statistical tests for nestedness are required to allow comparison of results from different studies.

Here we present FALCON – a free software package that allows the user to easily compute several measures of nestedness and associated statistical significances based on a selection of null models. FALCON stands for “Framework for Adaptive ensembLes for the Comparison Of Nestedness”. FALCON operates on any form of bipartite interaction data represented as a matrix of associations and is set up to be deliberately ‘blind’ to the source and interpretation of input data. FALCON is based on the assumption that nestedness is a general statistical property of matrices and therefore its measurement should be independent of context or interpretation. FALCON calculates nestedness as a statistical property of a matrix, by returning the nestedness score for the most-nested configuration of the input matrix. Since calculating statistical significance of nestedness scores can be computationally demanding, involving generation of a large ensemble of matrices from a null distribution, FALCON uses a novel “adaptive ensemble” method to improve efficiency by using the minimal ensemble size sufficient to give robust statistics.

Several software packages for calculating nestedness already exist – including ^1, 11, 17– 21^, but these are subject to various factors which make the direct comparison of different nestedness measures and the statistical interpretation of returned values difficult to achieve. Several nestedness measures are handled by packages which deliver a single measure, making the comparison difficult. Some are specific to a particular operating system. Some do not make the source codes available for re-implementation, reducing confidence in their outputs and prevent future extensions. Two packages for the R statistical programming language, bipartite ^19^ and vegan ^21^, together contain functions for several nestedness measures and associated null models, as well as many other tools for analysis of bipartite ecological networks. However, these packages offer no obvious implementation of significance testing (the principal method for reporting results of nestedness analyses) and they also lack several nestedness measures which have been recently developed. FALCON is designed to address these deficiencies, enabling the calculation of nestedness and statistical significance by using a variety of measures and null models, with open source code provided for several platforms.

The FALCON package is available for three commonly used numerical analysis platforms: MATLAB, Octave and R. MATLAB ( http://www.mathworks.co.uk/products/matlab/) is a commercial software platform, while Octave ( https://www.gnu.org/software/octave/) and R ( http://www.r-project.org/) are both freely available open source platforms. FALCON can be freely downloaded on Github ( http://github.com/sjbeckett/FALCON) or figshare ^22^ and all code is open and accessible. A guide to downloading, installing and running FALCON accompanies the code. This document describes the assumptions on which FALCON is based, how it calculates nestedness and statistical significance, gives details of the adaptive ensemble method used to improve computational efficiency and provides a case study to demonstrate its usage and outputs.

## 1 What is nestedness?

**Figure 1.**
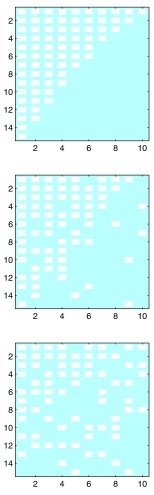
Perfectly nested, weakly nested, and randomly connected matrices. White squares indicate connections between two kinds of entity arranged in rows and columns.

Nestedness is a statistical property of bipartite interaction data presented in matrix form. In a perfectly nested matrix, the entries in each successive row are a strict subset of those in the previous row, while the entries in each successive column are a strict subset of those in the previous column ( Figure 1). Interpretation of nestedness depends on context.

The concept of nestedness was first described in studies on how species distributions varied between sites ^23– 25^, and later defined quantitatively as measuring the ‘amount of order/disorder’ in matrices representing the presence/absence of species in island communities ^1^. Used in this way, nestedness is calculated from a matrix of presence-absence data where rows are species and columns are sampling sites along some environmental or spatial gradient. A perfectly “nested” matrix (see Figure 1) would be achieved when the set of species present at each site along the gradient is a subset of the species present at the previous site. Since then, the concept of nestedness has been extended in various directions; see ^26^ for an historical overview of the nestedness concept. Nestedness has continued to be applied to spatial patterning (e.g. ^27^) and has been linked with *β*-diversity ^28^, but has also been applied to study mutualistic or antagonistic species-species interactions ^29, 30^, species-time relationships for a single site ^31^, and several other types of bipartite networks ^9, 10, 32– 35^. For pairwise interactions (e.g. plant-pollinator or host-parasite systems), nestedness has been interpreted as placing species along a gradient of generalism-specialism in the number of partners they interact with; in this context, perfect nestedness is achieved when species within each class are ordered such that the interaction set (set of partners) for each species is a strict subset of that of the next species, and the most generalised species of one class interact with the most specialised species of the other class.

Nestedness is calculated from a biadjacency matrix representing pairwise interactions between two kinds of entity (one represented by rows, the other by columns). The order of rows and columns for a biadjacency matrix is arbitrary with respect to connectivity; rows and columns can be permuted without affecting the underlying topology of the interaction network. Any non-arbitrary ordering of rows and columns in the matrix representation necessitates the use of additional contextual information to specify which order rows and columns should take. While some datasets may suggest a “natural” ordering to rows and columns in the matrix representation of data (e.g. when one of the dimensions represents an environmental/spatial/temporal gradient), for many applications of nestedness there is no natural ordering (e.g. species interactions).

As stated above, we consider that nestedness should be a context-free metric, so that it can be applied to data without requiring any supplemental information on row/column ordering. This assumption implies that the ordering of rows and columns should not affect the measurement of nestedness. While some nestedness measures are insensitive to row/column ordering, several of the most commonly used measures are highly sensitive to ordering, introducing indeterminacy to the quantification of nestedness when rows/columns are ordered arbitrarily. To avoid this indeterminacy and return a single robust nestedness score for a given input matrix, FALCON can sort the rows and columns such that nestedness (however calculated) is maximised. Since re-ordering rows/columns in a matrix representation does not alter the structural information (node adjacency) of the underlying data, this re-ordering is a reasonable approach and makes the measurement of nestedness more consistent.

## 2 Measures of nestedness in FALCON

Nestedness is most commonly calculated for binary data representing presence/absence of an interaction between two entities, but can also be calculated for weighted data that indicate the strength of the interaction. The methods used to calculate nestedness vary depending on whether binary or weighted interaction data are provided. The nestedness measures available in FALCON are shown and briefly described below and in Table 1; further details are given in Appendix A.

**Table 1.** Nestedness measures available in FALCON.

Name	Shorthand	Binary/ Weighted	Brief description	Reference
Nestedness based on overlap and decreasing fill	NODF	Binary	Pairwise row and column comparisons	[ 36]
Manhattan distance	MD	Binary	Sum of row and column indexes of connections	[ 37]
Nestedness temperature calculator	NTC	Binary	Difference from an ‘isocline of perfect order’	[ 1] [ 21]
Johnson, Domínguez- García & Muñoz	JDM	Binary	Measure of dissassortivity using configuration model	[ 38]
Discrepancy	BR	Binary	Difference from a ‘maximally packed’ matrix	[ 2]
Weighted NODF	WNODF	Weighted	Weighted version of NODF	[ 20]
Spectral radius	SR	Both	Maximum real eigenvalue of adjacency matrix	[ 11]

The nestedness measures considered here are not trivial variations upon each other, but differ significantly in their derivations. However, some similarities can be drawn. Spectral radius (SR) ^11^ and the measure of Johnson, Domínguez-García, & Muñoz ^38^ (JDM) are invariant to the ordering of rows and columns in the network and are calculated using the adjacency matrix of the network. On the other hand, discrepancy (BR) ^2^, Manhattan distance (MD) ^37^ and nestedness based on overlap and decreasing fill (NODF) ^36^ are all sensitive to row/column ordering and are maximised when rows/columns are ranked by degree. The nestedness temperature calculator (NTC) ^1, 21^ involves sorting of rows and columns against the ‘isocline of perfect order’ (see Figure 5) such that it maximises connections above the isocline and minimises connections below the isocline. BR is similarly calculated relative to an idealised ‘maximally packed’ matrix. NODF is found through pairwise comparisons of overlap between subsequent rows and columns, whilst MD is found by assigning a weight to each connection as a sum of it’s row and column indexes. The measures also differ in how nestedness is scored; the degree of nestedness in a network increases with increasing measure score for JDM, NODF and SR, but with decreasing measure score for BR, MD and NTC.

## 3 Comparison of nestedness scores

Nestedness is strongly sensitive to the size (number of rows and columns) and fill (number of non-zero entries) of the input matrix ^17^. This is problematic in practical terms, since we often wish to compare nestedness of matrices that differ in these basic properties; in fact, cases where we compare empirically derived matrices with identical size and fill are an exception. Thus comparison of absolute values of nestedness metrics is not informative and may be misleading. To compare nestedness of matrices with differing size and fill, observed nestedness should always be interpreted in the context of a null distribution of matrices with similar properties. Measuring observed nestedness relative to expected nestedness derived from a null distribution of similar matrices allows determination of both effect size (e.g. as a *z*-score, which is commonly used to compare different nestedness schemes ^26, 39^) and statistical significance (e.g. as a *p*-value giving the expected frequency of the observed score in the null distribution). This approach necessitates choice of a suitable null model and generation of a distribution of random matrices drawn from it.

In the present context, a null model is a method for creating a distribution of matrices that conserves some properties of the input matrix while varying other properties at random ^40^. We continue the “context-free” approach in our treatment of null models; to allow comparison of nestedness across different scenarios, a good null model should not make assumptions about the mechanisms by which data were generated, but treat the matrix as an independent data structure. However, to be comparable to the input matrix, null matrices must conserve some key matrix properties (such as size and fill) on which nestedness depends. The null models available in FALCON are given in Table 2; further detail is given in Appendix B. FALCON includes some of the more popular null models from the literature, alongside some additional null models that we feel can be useful. Null models vary in whether the original data is binary or quantitative, and in which properties of the original input matrix are preserved.

**Table 2.** Null models in FALCON.

Name	Description	Binary/ weighted	Conserved features	Reference
SS	Shuffles positions randomly	Binary	Shape, Fill	[ 11]
FF	Permutations of structure with same node degrees	Binary	Shape, Fill, Degree	[ 41]
CC	Some structure is preserved, rest is shuffled	Binary	Shape, Fill	
DD	Determined probabilistically by node degree	Binary	Probabilistic Degree	[ 3]
EE	Determined probabilistically by fill	Binary	Probabilistic Fill	
Binary Shuffle	Order of weighted links is swapped	Weighted	Binary positions, weights	[ 11]
CRT	Random weights where row totals conserved	Weighted	Binary positions, row totals	
CCT	Random weights where column totals conserved	Weighted	Binary positions, column totals	
RCTA	Average of conserve row totals and column totals	Weighted	Binary positions	

## 4 How FALCON works

### 4.1 Inputs and outputs

FALCON requires several inputs:

- an input network in the form of a bipartite matrix
- whether binary or quantitative nestedness should be investigated (quantitative matrices can be analysed using binary measures)
- whether to sort rows and columns to maximise nestedness score
- which nestedness measures should be used
- which null models nestedness should be tested under
- whether the ensemble of null models should be created with a fixed number or adaptively chosen
- whether or not to plot the distributions of nestedness scores

Output is returned to the user in the form of:

- the most nested configuration of the input matrix
- the nestedness measure(s) of the input matrix
- the expected value of nestedness under the null model(s) (as the mean measure of matrices created in the ensemble)
- the number of ensemble members used to calculate significance in each null model
- the statistical significance of the nestedness of the input matrix against each null model as a p-value
- the standard deviation and sample *z*-scores of the measure in the ensemble as well as other properties.

### 4.2 What FALCON does

**Figure 2.**
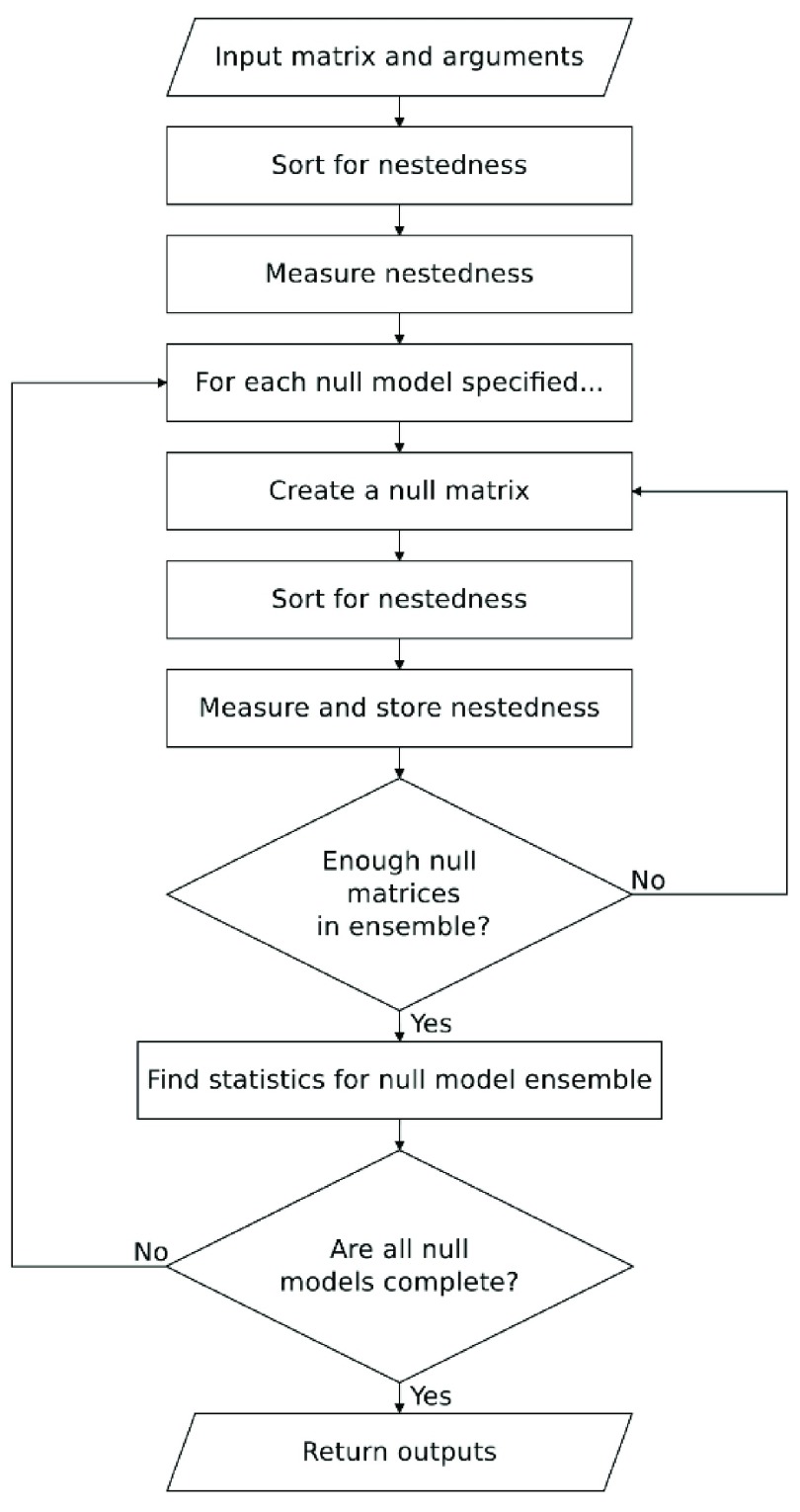
FALCON algorithmic procedure.

FALCON follows the process shown in Figure 2. First, it sorts the user input matrix into a maximally nested configuration and removes any empty rows/columns before finding the nestedness of this matrix using the users chosen measures. Then, FALCON goes through each of the user specified null models one by one, creating an ensemble of null matrices according to the rules of each null model. Each null matrix is then sorted and measured by each of the chosen nestedness measures. Thus, for each null model, nestedness measures are calculated for each of the null matrices in a single null ensemble, enabling direct comparison of results. The size of the null ensemble is determined by the input choice of using either the fixed or adaptive ensemble size (see Section 4.5). Statistics are computed from the measures found in the null ensemble (and the direction in which that nestedness measure is calculated), before the next null model ensemble is instantiated. Once all null models have been computed, the results are returned to the user.

### 4.3 Direction of increasing nestedness

**Figure 3.**
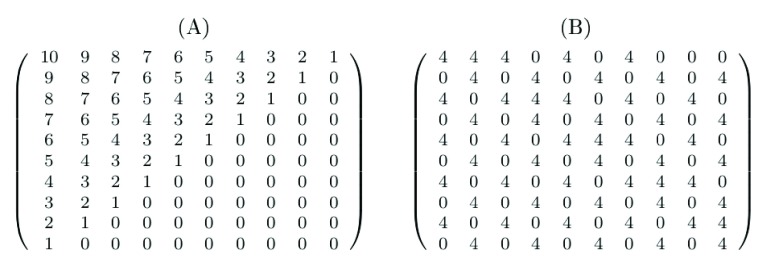
( **A**) Weighted nested matrix. ( **B**) Weighted non-nested matrix (checkerboard). Both ( **A**) and ( **B**) have 55 non-zero elements that sum to 220.

For different nestedness measures, increasing scores can represent either increasing or decreasing nestedness as discussed in Section 2. FALCON initially determines whether a higher measure score is related to greater nestedness (or vice versa) in the chosen measure by comparing the scores returned for a highly nested network (see Figure 3A) and a highly non-nested network (a weighted checkerboard configuration; Figure 3B), for which the fill (number of non-zero elements) and element sums are equal. The direction of increasing nestedness for a given measure is used during calculation of statistical significance. This method of determining direction each time the algorithm runs is included to allow easy extensibility; if a new measure is added, FALCON will automatically determine which direction indicates increasing nestedness.

### 4.4 Initial sort

For efficiency, FALCON is set up to initially sort the input matrix by row and column degrees for calculation of BR, MD and NODF, retains this sorted configuration for calculation of JDM and SR, and subsequently re-sorts for NTC in order to find the maximal nestedness of a binary matrix. For quantitative data, FALCON uses the same methods as for binary interactions, but also utilises weight data to break symmetry when two rows (columns) have the same degree; in this case, the row (column) which has greater values for most overlapping elements is ranked highest. Where two or more rows (columns) share the same degree and most overlapping elements, the rows (columns) are ranked according to the total sum of row (column) elements. This sorting does not affect the underlying topology or the relationships in the data. FALCON also allows the user to decide if any sorting is performed, enabling the “context free” assumption to be relaxed (e.g. for investigation of gradient-based nestedness ^39^).

### 4.5 Size of null ensemble

FALCON uses a bootstrap method to calculate the statistical significance of a given nestedness score, since the true null distributions of the test statistics are not known. The ensemble size used for this calculation can either be fixed or calculated adaptively by FALCON to improve computational efficiency and reduce undersampling effects. Note that the strongest significance that can be assigned is $P<\frac{1}{N}$ where *N* is the ensemble size.

***Fixed.*** The number of null matrices used to make up the ensemble is fixed by the user. This method is effective providing that the ensemble is large enough to have statistical power; the larger the ensemble, the more power the test has and the closer the answer will be to the *p*-value for the (unknown) true null distribution. However, it is not obvious how large the ensemble needs to be; in the literature, amongst others ^30^, use 1,000 null models in their ensembles, whilst ^12^ use 10,000, and ^6^ use 100,000. A large number of different null matrix configurations are possible for a given input matrix and we may wish to avoid undersampling ^42^; however, at the same time very large ensembles can make the calculation of significance computationally intractable.

***Adaptive.*** FALCON includes a mechanism for adaptive determination of ensemble size. This is intended to ensure robust statistics are achieved, avoiding concerns about undersampling or oversampling ^42^, while minimising computational load. The adaptive method works by creating two ensembles in parallel using the same null model. Starting with a minimum ensemble size of 500 in each group, the ensembles are expanded until they show similar statistical properties. This condition is met when the null hypothesis (both ensembles come from the same distribution) of a Mann-Whitney U-test cannot be rejected at 10% significance. When this occurs, it suggests each group represents a good sample of the underlying distribution, and the two groups are combined to form a single null ensemble used to calculate final statistics. The expansion of the size of the ensemble has an upper limit of 100,000 members in case the null hypothesis is always rejected. The adaptive ensemble methods balances statistical precision with computational efficiency; we conservatively use 1,000 as a minimum final ensemble size such that a *p*-value as low as 0.001 can be assigned.

### 4.6 Output statistics

***p-value.*** The *p*-value is the probability that a matrix drawn from the null distribution will be more nested than the input matrix. Low values ( *p →* 0) indicate that the input matrix is highly nested relative to the null distribution; commonly a threshold of *p ≤* 0.05 or *p ≤* 0.01 is used to denote a statistically significant level of nestedness. Here *p* is calculated by counting the frequency of matrices in the null ensemble that are more nested than the input matrix; for cases where no member of the null ensemble is more nested than the input matrix we conservatively assign $P<\frac{1}{N}$ where *N* is the ensemble size.

***Normalised Temperature.*** The normalised temperature is inspired by the *τ*-Temperature ^37^. It describes the relationship between the nestedness measure found for the input matrix and the expected nestedness measure derived from the null model ensemble. It is described as:

$$T\;=\;\frac{Measure}{<Measure>}\;\;\;\;\;(1)$$

where *< Measure >* denotes the expected value. In simple terms, the normalised temperature indicates whether the input matrix is more or less nested than the expectation for a null distribution of similar matrices. Where the measure gives increasing scores with increasing nestedness, *T* > 1 indicates greater-than-expected nestedness. Where the measure gives decreasing scores with increasing nestedness, *T* < 1 indicates greater-than-expected nestedness.

***Mean.*** The mean average of the set of nestedness measures found for each of the ensemble members is returned.

***Standard Deviation.*** The standard deviation ( *σ*) of the set of nestedness measures found for each of the ensemble members is returned.

***Sample z-score.*** The *z*-score, or standard score, is calculated as the difference between the nestedness measure and its expected value divided by the standard deviation of the sample:

$$z\;=\;\frac{Measure\;-<Measure>}{\sigma }\;\;\;\;\;(2)$$

It is a measure of the number of standard deviations the nestedness measure of the input matrix is above the expected value. Hence, the way it should be interpreted, as with the normalised temperature, depends on whether nestedness increases with increasing measure score.

## 5 FALCON usage - case study

To demonstrate FALCON we analyse nestedness analysis in a bipartite network representing the hashtags used by a sample of Twitter users. Data were collected using the Twitter API ( https://dev.twitter.com/docs/api) by searching for tweets including the hashtag “#IPCC” in the time period 21st September 2013 – 5th October 2013. A list of all hashtags used by all users found in the search dataset was then used to create a binary bipartite adjacency matrix for users and hashtags. This was then sampled to create a smaller matrix used for this case study by including each row/column with probability of 1.1 and removing any empty rows/columns. The resulting matrix was stored in a comma-separated file called ’IPCC_HTuse_10_10_1_53x27.csv’.

**Figure 4.**
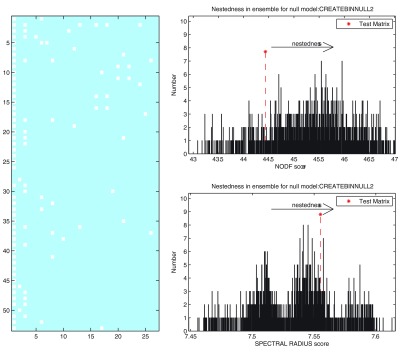
Example output from FALCON. ( **A**) the nested arrangement of the UserHashtag data. ( **B**) Distribution of NODF scores found for an ensemble of FF null models generated for the UserHashtag network ( **C**) distribution of spectral radius scores found from the same null matrix ensemble. The asterix marks the nestedness score of the input network.

**Figure 5.**
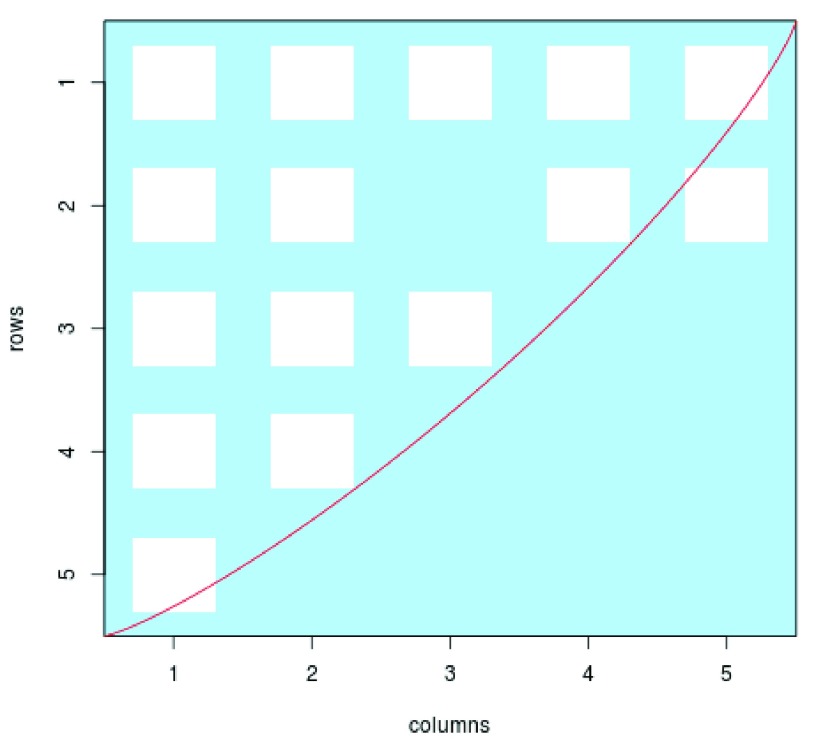
The NTC measure is based on an isocline of perfect order. Here there are two ‘surprises’.

The box below shows the command sequence used to perform a binary nestedness analysis using FALCON in MATLAB. The first line reads in the “.csv” datafile, which includes row and column headers. The second line extracts the adjacency matrix from the imported data. The third line runs FALCON, using two binary nestedness measures (NODF and SR) and two null models (CC and FF, i.e. nulls 2 and 3) using the adaptive solver and displaying histogram plots. The fourth line plots the input matrix in its most nested configuration, as determined by FALCON. The nested configuration of the matrix and output histograms from significance testing are shown in Figure 4, whilst Table 3 shows an example output from the significance testing. Further examples for use of FALCON in R are given in supporting information accompanying the software.

**Table 3.** Example output from FALCON showing sample statistics for the UserHashtag network using the operations in the box above. Nestedness statistics were computed for the FF and CC null models using the NODF and spectral radius measures.

Null model	FF		CC	
Nestedness measure	NODF	SR	NODF	SR
Measure	44.4339	7.5552	44.4339	7.5552
Ensemble Size	1000	1000	1000	1000
Mean	45.3207	7.5346	29.9048	6.4755
Standard Deviation	0.8357	0.0336	3.5600	0.2136
*z*-score	-1.0612	0.6123	4.0812	5.0550
*p*-value	0.8490	0.2480	<0.001	<0.001
Normalised Temperature	0.9804	1.0027	1.4858	1.1667

1 >> UserHashtag = importdata(‘IPCC_HTuse_10_10_1_53x27.csv’)
2 >> data = UserHashtag.data;
3 >> output = PERFORM_NESTED_TEST(data,1,1,{’NODF’,
 ’SPECTRAL_RADIUS’},[2,3],[],1)
4 >> MATRIXPLOT(output.NestedConfig.DegreeMatrix)

## 6 Summary

In this paper we have presented FALCON, a software tool for reliable and efficient calculation of nestedness (and associated effect size and statistical significance) based on a selection of popular nestedness measures and null models used in the literature. FALCON treats nestedness purely as a statistical property of a bipartite matrix and removes any form of interpretation or contextual information from the analysis. This enables FALCON to be used to compare nestedness across a wide variety of application areas, noting that the concept of nestedness has already spread from its origin in island biogeography to include species-species interactions and other scenarios, and is likely to find further applications in other domains. The contribution of FALCON is to enable easy cross-comparison of observed nestedness using different nestedness measures and null models. We hope that this functionality will allow greater methodological uniformity and comparability of studies of nestedness. We are in the process of performing a large comparison study of nestedness metrics using FALCON (Beckett and Williams., in preparation). Uniformity of measurement and comparability of empirical results is an important preliminary step that must be achieved to enable understanding of the mechanistic basis and ecological (and otherwise) implications of nestedness. We hope that FALCON will be of use to other researchers and help illuminate this intriguing property of bipartite networks in many natural systems.

## 7 Software availability

### Software access

The FALCON software package is available from Github: http://github.com/sjbeckett/FALCON

### Source code as at the time of publication

https://github.com/F1000Research/FALCON

### Archived source as at the time of publication

http://dx.doi.org/10.6084/m9.figshare.999117 ^22^

## 8 Appendix A: Detailed description of nestedness measures in FALCON

### 8.1 Binary

***NODF.*** The nestedness measure based on overlap and decreasing fill (NODF) was first described by ^36^ and has since become one of the most popular methods for describing the nestedness of a matrix. NODF can be found as:

$$NODF\;=\;\frac{N_{col}+N_{row}}{\frac{\left(c(c-1\right)}{2}+\frac{\left(r(r-1\right)}{2}}=\frac{2(N_{col}+N_{row})}{c(c-1)+r(r-1)}\;\;\;\;\;(3)$$

Here *N _col_* and *N _row_* are scores found by pairwise comparison of rows and columns, *c* is the number of columns, and *r* is the number of rows. *N _col_* is found as the sum of scores from pairwise comparisons of each column against all columns to its right. If both columns have the same degree, then the score is zero. If they have different degrees, the score is the percentage of elements in the second column which also appear in the first column. *N _rows_* is found similarly for pairwise comparisons of each row against all rows below it. The sum of *N _col_* and *N _row_* is then normalised by the total number of pairwise comparisons. Values for NODF are between 0 (zero nestedness) and 100 (perfect nestedness). If the input matrix is first sorted to maximise nestedness by rank ordering rows and columns by degree, the form of NODF known as *NODF _MAX_* is found ^43^.

***τ-Temperature and Manhattan distance.*** The *τ*-Temperature ^37^ is a nestedness measure based on relative distances between matrix elements. Unlike other distance-based measures (such as NTC ^1^ and its better described successors BINMATNEST ^17^ and AININHADO ^18^), the *τ*-Temperature does not use genetic algorithms to sort the data. The *τ*-Temperature is found by measuring the Manhattan distance *D* of the network matrix. This is the sum of the row and column indexes of all of the matrix elements *A _ij_* that are filled:

$$D=\sum_{A_{ij}>0}\left(i+j)\;\;\;\;\;(4\right)$$

Manhattan distance is lower in more highly nested networks, since rows and columns can be shuffled so that many of the elements appear in upper-left positions where row and column indices are low. Once *D* is found, a null model is chosen (cf. Section 3) and an ensemble of null matrices are created. By finding the mean average Manhattan distance from the ensemble, denoted < *D _rand_* >, *τ*-Temperature can be calculated as:

$$\tau =\frac{D}{<D_{rand}>}\;\;\;\;\;(5)$$

Values *τ* > 1 imply that *D* is greater than < *D _rand_* > and the network is less nested than expected for a network with the properties defined in the null model. *τ* is better described as a test statistic of the Manhattan distance, than as a measurement of nestedness itself.

***JDM Nestedness.*** The nestedness measure described in ^38^, here termed JDM after author initials, treats nestedness as a measure of dissassortativity between the nodes, i.e., negative correlation between row and column degrees for non-zero elements of the input matrix. Their measure calculates the overlap (as the sum of the elements in the squared adjacency matrix which shows the minimum number of length two paths needed to connect any two nodes) of the input matrix and normalises it by the expected nestedness of the configuration model (a random graph with the same empirical degree distribution as the input network) and thus discounts the effect of degree heterogeneity. This nestedness score is unbounded, but when close to 1 it indicates that the matrix represents an uncorrelated random network. Unadjusted nestedness $\tilde{\eta }$ is calculated using the adjacency matrix *a* formed from the input bipartite matrix with *r* rows and *c* columns, where *D* is the node degrees in the adjacency matrix:

$$\tilde{\eta }=\frac{1}{{\left(r+c\right)}^{2}}\sum_{i}^{\left(r+c\right)}\sum_{j}^{\left(r+c\right)}\left(\frac{a_{i\;j}^{2}}{D_{i}D_{j}}\right)\;\;\;\;\;(6)$$

Nestedness of the configuration model *η _conf_* can be calculated as:

$$\eta_{conf}=\frac{r\left(\frac{\sum_{i}^{r}k_{i}^{2}}{c}\right)+c\left(\frac{\sum_{j}^{c}d_{j}^{2}}{r}\right)}{\left(\frac{\sum_{i}^{r}k_{i}}{c}\right)\;\;\left(\frac{\sum_{j}^{c}d_{j}}{r}\right)\;\;{\left(r+c\right)}^{2}}\;\;\;\;\;(7)$$

which can also be written as:

ηconf=  r<k2>c+c<d2>r<k><cd>r(r+c)2     (8)

where *k* are the row degrees and *d* are column degrees in the bipartite matrix. This leads to the normalised measure of nestedness for bipartite networks defined by ^38^ as:

$$\eta_{bip}=\frac{\tilde{\eta }}{\eta_{conf}}\;\;\;\;\;(9)$$

***Nestedness Temperature.*** The original nestedness temperature calculator (NTC) ^1^ was vaguely described and therefore difficult to reimplement, leading to several subsequent variations utilising similar underlying principles ^15, 17, 18, 21^. Here we have recoded the nestedtemp function from the R package *vegan* ^21^. The nestedness temperature for an input matrix is based on the ‘isocline of perfect order’, a curve drawn from the lower-left corner of the matrix to the upper-right, with curvature defined by matrix fill (see Figure 5). Row and column orderings are then permuted using a genetic algorithm to maximise the number of connections above the isocline and minimise connections below the isocline. The number of connections which violate these rules, termed ‘surprises’, is then calculated and normalised to give a score between 0 (highly ordered) and 100 (highly disordered).

***Discrepancy.*** Discrepancy ^2^, here denoted BR, quantifies nestedness as the difference between the input matrix and a perfectly nested matrix of the same dimensions and fill. The duplicate matrix *P* has the same row degrees as the input matrix, but the 1s in each row are pushed as far to the left as possible (ignoring the effect this has on underlying network topology). The discrepancy is then found by subtracting the input matrix from this perfectly nested matrix and counting the number of 1s that remain – the number of differences between *P* and the input matrix. By treating columns instead of rows an alternative perfectly nested comparator matrix *P′* can be formed by pushing the 1s in each column of the input matrix as far to the top as possible – from which a different discrepancy score can be found. Here we modify the original method of ^2^, which looks at discrepancy only in respect to *P*, and instead define discrepancy as the minimum of the individual discrepancy scores found from *P* and *P′*, to remove any bias towards row or column nodes.

### 8.2 Quantitative

***WNODF.*** The weighted NODF measure, WNODF ^20^, uses a similar algorithm to NODF, but is designed for use on quantitative rather than binary networks. In addition to asking which pairs of rows/columns are subsets of one another, WNODF utilises weight information by also requiring that the preceding row/column has greater values in the overlapping elements. In effect, WNODF is a stricter version of NODF; the maximum WNODF score that can be achieved for a quantitative matrix is equal to the NODF score for the binary matrix.

### 8.3 Both

***Spectral Radius.*** The spectral radius (SR) is defined as the absolute value of the maximum real eigenvalue from the adjacency matrix of a given input bipartite matrix. SR was proposed as a nestedness measure by ^11^ and can be applied to both binary and quantitative matrices.

## 9 Appendix B: Null models available in FALCON

### 9.1 Binary

***Swappable-Swappable (SS).*** The “swappable rows, swappable columns” (SS) null model conserves matrix dimensions (numbers of rows and columns) and fill. It is similar to ‘test one’ in ^11^, which works by shuffling elements at random within the matrix; however, it differs in that degenerate matrices (those containing rows/columns with no connections) are not permitted.

***Fixed-Fixed (FF).*** The “fixed rows, fixed columns” (FF) null model conserves dimensions, fill and degree distribution of the original matrix. It is the most strict null model we consider here and is known to suffer from Type II errors (i.e. a failure to detect nestedness) ^30^. We use the curveball algorithm ^41^ to generate null matrices of this type. It does this by iteratively choosing pairs of rows at random, compiling a list of column indices which contain filled elements in one but not both of the two rows. This list of column indices is then randomly permuted and reassigned to the two rows corresponding to the number of unique positions belonging to each of the original rows. It should be noted that the Manhattan distance is invariant to these permutations.

***Cored-Cored (CC).*** The “cored rows, cored columns” (CC) null model conserves dimensions and fill as in the SS null model, but also conserves some of the core structure found in the observed input matrix. It is found by performing a total of *M × N* trial-swaps on the *M × N* input matrix, where two matrix elements are randomly chosen and their values can be swapped only when this does not reduce the corresponding row or column degrees to zero. This ensures that the size structure is conserved and preferentially preserves specialist interactions within the network. The removed elements are then randomly reassigned to the remaining empty spaces to preserve matrix fill.

***Degreeprobable-Degreeprobable (DD).*** The “degreeprobable rows, degreeprobable columns” (DD) null model first described by ^3^ has subsequently been a popular choice for application to species-species nested comparisons. Matrix elements are probabilistically determined depending on the degree distribution of the rows and columns of the initial matrix as:

$$p_{ij}=\frac{1}{2}\left(\frac{d_{j}}{r}+\frac{k_{i}}{c}\right)\;\;\;\;\;(10)$$

where *p _ij_* is the probability of assigning a 1 to the ith row and jth column of the null matrix, *d _j_* is the column degree of the jth column, *k _i_* is the row degree of the ith row and *r* and *c* are the respective number of rows and columns. Due to the stochastic nature of this null model its output matrices will vary in size and fill.

***Equiprobable-Equiprobable (EE).*** The “equiprobable rows, equiprobable columns” (EE) null model is probabilistic and assumes that the probability of a connection occurring between two nodes is related to the number of total connections in the input matrix. Hence for an input matrix with fill *M*, *r* rows and *c* columns, the probability of a connection being present between two nodes is $p_{ij}=\frac{M}{r\times c}$. Due to the stochastic nature of this null model its output matrices will vary in size and fill. It is the least strictly defined null model we consider here and is known to suffer from Type I errors (a tendency to falsely detect nestedness) ^30^.

### 9.2 Quantitative

***Binary Shuffle.*** This null model was employed by ^11^ and conserves the entire binary structure of the input matrix and the values of the elements in the matrix, but shuffles the order of these values randomly across the binary structure.

***Conserve Row Totals (CRT).*** This null model conserves binary structure and the row sum totals, but the values of the elements on each row are changed such that each connection in the row is assigned a random proportion of the row sum total.

***Conserve Column Totals (CCT).*** This null model conserves binary structure and the column sum totals, but the values of the elements in each column are changed such that each connection in the column is assigned a random proportion of the column sum total.

***Row Column Totals Average (RCTA).*** Both of the two above null models conserve information related to either the rows or the columns, giving this property precendent over that of the other entity. We also introduce the Row Column Totals Average (RCTA) null model which uses the average of a single null model made from each of the CCT and CRT null models. As information from both rows and columns is utilised in the creation of this null model it may better fit with the context free ethos of nestedness we pursue than either of CRT or CCT alone.
