# Supplementary material for: FALCON: a software package for analysis of nestedness in bipartite networks
Source: F1000Res. 2014 Aug 6;3:185. [Version 1] doi: 10.12688/f1000research.4831.1 (PMC4244763; doi:10.12688/f1000research.4831.1)
Supplement: Peer review history

# Peer review: Referee response for version 1

**DOI:** 10.5256/f1000research.5156.r6467

**Published:** 2014-11-19

**Authors:** Justin R. Meyer

**Affiliations:** 1 Systems Biology Department, Harvard Medical School, Boston, USA

This is a great, easy-to-use, and comprehensive piece of software. I am eager to begin to use it myself and to send it to colleagues who are reluctant to run the other nestedness software. The paper is also very well-written making this field more accessible. As the authors state, many forms of biological data are organized in bipartite networks and show nested pattern. With such a software platform and clear manuscript, I hope more similarities across biological systems will be recognized and appreciated, perhaps even promoting collaborations and new theory. My one suggestion is for the authors to provide a more thorough description of what null models should be used with which data structures, sampling procedures, biological processes, ecs. This would aid the cross-system comparisons and users from finding spurious patterns.

I have read this submission. I believe that I have an appropriate level of expertise to confirm that it is of an acceptable scientific standard.

# Peer review: Referee response for version 1

**DOI:** 10.5256/f1000research.5156.r5722

**Published:** 2014-08-12

**Authors:** C. Patrick Doncaster

**Affiliations:** 1 Centre for Biological Sciences, University of Southampton, Southampton, UK

The paper introduces a new freeware package designed to collate existing measures of nestedness in biological samples. This is a useful capability because several different methods exist for quantifying parameters and testing significance. The paper describes a novel and efficient method for sampling the null distributions that calibrate the significance of nestedness scores. The package should interest anyone working with biological networks of bipartite data, and is conveniently available for open-source platforms R and Octave as well as MATLAB.

I have no major concerns about the conception, execution or description of the paper. It appears to me well motivated, technically sound and written with considerable clarity. The case study provides a useful illustration, though is under-developed as I will detail below and perhaps represents a missed opportunity. I found the program itself easy to use and really useful for exploring alternative measures of nestedness

The manuscript title answers to the content of the paper. The Abstract provides a clear summary, though its impact would benefit from removal of repetition. An abstract of 150-words in a single paragraph could state concisely what the need is, and how the package addresses it.

The Introduction could usefully explain the meaning of nestedness (currently in the following section) before briefly reviewing alternative methods of quantification. That review should attempt some categorisation of existing methods in one paragraph, and examples of their applications, before describing their various limitations in a following paragraph. Then say how FALCON addresses these issues.

The paper provides a clear description of the concept of nestedness and its interpretation, which is not an easy task. It would help to define ‘bipartite’ in the context of the Fig.-1 matrices, as the two dimensions that make up the matrix columns and rows. Table 1 gives shorthand codes for the various nestedness measures, though only some of these correspond to the codes for calling the measures in the program (Table 2 of the instruction guide). It would help to have a closer correspondence of shorthand with code of measures, and with the subtitles in Appendix A. Likewise for null models and Appendix B.

The Section-5 case study should take the opportunity to show readers how they can find interesting patterns in datasets by calculating nestedness, using sensible measures and calibrations. For this particular example, it would help to clarify the context by indicating that ‘IPCC’ stands for either ‘Intergovernmental Panel on Climate Change’ or ‘Independent Police Complaints Commission’. It’s not clear to me whether this hashtag was chosen purposefully to sample such different populations of Twitter users, and if so how one might interpret nestedness in the aggregate population. Any case study serves its purpose only if it is followed through all the way from motivation through to extraction of qualitative meaning from the quantitative analysis. Thus the text needs to interpret Figure 4 qualitatively, explaining the choice of measure and null model for these data, and resulting nestedness score such that the reader can see how nestedness might reveal interesting pattern in the data. It would be good to have the case-study dataset made available for users to try out for themselves.

In the case study, the textual explanation of the command box says that the program uses three null models, but as far as I can see it calls only two: FF and CC. Then, confusingly the output graphs of null distributions show only the first one: CREATEBINNULL2. Actually, the second one looks more interesting according to Table 3. These steps from input to output need more explanation. Particularly for Fig. 4 and Table 3, interpretation is not helped by the lack of correspondence between names used for shorthand (e.g., ‘SR’, ‘FF’) and code (e.g., ‘SPECTRAL_RADIUS’, ‘CREATEBINNULL2’). If correspondence is not possible in the program itself, it would help at least to have clearer titles for graphs and tables.

When using the package, I thought that the instruction guide would benefit from some tidying up. For example, it would help to tabulate all possible alternative values for each of the seven options. Null models should be presented after measures, since a null only has meaning as the baseline against which to calibrate a measure. For the R version of the program, an example script is provided to illustrate capability (examplescript.R). Its ‘TEST 2’ loads a .csv file containing a matrix suitable for nestedness analysis, but omits the specification ‘header = FALSE’. As a result, it reads the first line of the file as a header, which was surely not the intention.

Specific points that merit attention:

 

1. Last paragraph of Section 1, say: “FALCON can sort rows and columns so as to maximise nestedness regardless of the method used for its calculation.”
2. When referring to specific published texts, remove the superscript, to say for example (Section 4.5): “in the literature, ref 30 amongst others use 1000 null models in their ensembles, whilst ref 12 use…” etc.
3. Figure 4 component parts need labels (A), (B), (C). Label the axes of the matrix, at least in the legend if not in the figure itself: presumably columns for users and rows for hashtags.
4. Table 3, avoid vertical lines in tables. The table would be easier to read if columns and rows were switched around. Left-justify text, and use a decimal tab to align numbers in a column.
5. Avoid the phrase ‘As stated above’ – reorganise to state only once.
6. Avoid ending a sentence in a verb. The verb imparts meaning to the sentence by describing the action, so giving it early facilitates the reader’s comprehension.

I have read this submission. I believe that I have an appropriate level of expertise to confirm that it is of an acceptable scientific standard.
